# A Longitudinal and Interdisciplinary Biodesign Internship Program for Biomedical Engineering Undergraduate Students Focused on Medical Device Innovation

**DOI:** 10.1007/s43683-025-00174-w

**Published:** 2025-02-18

**Authors:** Lauren M Maloney, Christopher Page, Michael Bielski, Annie Rohan, Wei Yin

**Affiliations:** 1 Department of Emergency Medicine, Stony Brook Medicine, Stony Brook, NY 11794 USA; 2 Department of Anesthesiology, Stony Brook Medicine, Stony Brook, NY 11794 USA; 3 Center for Biotechnology, Stony Brook University, Stony Brook, NY 11794 USA; 4 School of Nursing, Stony Brook University, Stony Brook, NY 11794 USA; 5 Henry P. Becton School of Nursing & Allied Health, Fairleigh Dickinson University, Teaneck, NJ 07666 USA; 6 Department of Biomedical Engineering, Stony Brook University, Stony Brook, NY 11794 USA

**Keywords:** Biodesign, Clinical needs, Innovation, Summer immersion program

## Abstract

**Challenge:**

The biomedical engineering (BME) capstone design courses are traditionally offered in students’ senior year. Students often feel underprepared for the hands-on biodesign and prototyping process. Also, capstone design projects are often provided by BME faculty, without students’ input in needs finding and screening.

**Novel Initiative:**

A longitudinal and interdisciplinary biodesign internship course sequence (program) was developed and offered. This internship course has three components. Part I is offered in the fall semester of students’ junior year, focusing on biodesign concept development and preliminary prototyping. Part II is offered in the spring semester of students’ junior year, focusing on clinical observation and needs finding. Part III is a 6-week summer immersion program, where students work directly with clinicians and industry mentors to convert a valid clinical need into a biodesign project and initiate the bio-innovation process. Successful summer projects can be carried forward into students’ senior year and become their senior design projects.

**Reflection:**

Since the start of this program in 2018, 47 students participated in the program, which accounted for approximately 20% of the total number of students. More than 75% of the projects developed in the biodesign internship program were successfully carried into BME senior design and involved more than 60% of BME students in their senior design process. Students were satisfied with their biodesign internship experience. Other products of this program include conference presentations, peer-reviewed journal publications, provisional patents, patents, and design competition awards.

## Challenge Statement

As biomedical engineering (BME) undergraduate education evolves to meet the ever-changing medical device landscape its graduates will find their place in, more programs have begun to incorporate clinical immersion experiences into their curriculums [1–3]. Clinical immersion has proven to be invaluable for BME students as they help students to see first-hand challenges of the clinical environment in which their devices must function and learn how to distill what clinicians actually need from what they report they want [1, 2, 4, 5]. Ideally, engineering students should be given opportunities to perform their own clinical needs finding within the clinical environment (as compared to the more traditional approach of being provided with faculty-driven research or clinical needs for their BME senior design capstone projects) [2, 5]. This could help the students to improve their interdisciplinary communication skills as well as their understanding of the constraints during clinical needs screening [1, 5]. As such, this teaching tip article describes the creation and evaluation of a longitudinal and interdisciplinary biodesign internship program which brings together undergraduate BME students, medical students, clinical mentors, and industry mentors, with the goals that (1) BME students can learn a shared language of medical device innovation with medical students via longitudinal exposure to the process; (2) both BME students and medical students can benefit from the interdisciplinary collaboration and near-peer mentoring experience; 3) BME students can learn about different aspects of the biodesign and innovation process from clinical and industry mentors, before they start their senior design.

## Novel Initiative

This program helped to (1) shift BME capstone design projects from faculty-centric to clinical needs-driven projects identified by students themselves, (2) facilitate interdisciplinary collaboration between medical students and BME undergraduate students, and (3) expand the students’ biodesign process from one year (senior year) to two years (junior and senior years). Two courses were created, one for BME juniors and one for medical students. The BME course was taught in three consecutive semesters, i.e., fall, spring, and summer during the students’ junior year. The new course developed for medical students was described in detail elsewhere [6].

The BME course (EXT488) series, or program, was developed and implemented in the BME undergraduate program at Stony Brook University, an ABET (Accreditation Board for Engineering and Technology) accredited program, as part of an NIH R25 grant. The program has been successfully offered to five cohorts (2019 – 2023), with one cohort beginning every fall semester, and each cohort staying within the program for 2 years. No more than 12 undergraduate BME students, selected from about 1/3 of the junior class (35-55) who apply, are invited to enroll in each entering cohort, based on their academic record, extra-curricular activities, performance in the previous classes with significant design components, recommendations from faculty, and a personal statement (selection decision is made by the EXT488 program director and clinical mentors).

### EXT488 Part I: Introduction to the Medical Device Innovation Process (Fall, Year 1)

In this one-credit course (1 hour lecture/meeting time, and 3 hours of after-class lab/research work per week), BME students begin by joining medical students for a series of seminar sessions to learn the process of medical device innovation (e.g., clinical needs finding and screening, market analysis, intellectual property consideration, prototyping practices, etc.) [7]. These sessions are led by clinical faculty with engineering or device innovation experience. A non-medical, more universal need is pursued by interdisciplinary small groups during the sessions to keep the focus on understanding the process itself, and not get distracted by deciphering medical minutiae. For example, a class favorite was solving the dilemma of how to evenly distribute toppings on movie theater popcorn. During these sessions, students begin to learn how to communicate among themselves by limiting their respective professional jargon, and instead, emphasizing the use of their new shared terminology of innovation. The final session consists of a product pitch, modeled after the TV show Shark Tank, thus pushing the teams to not only communicate effectively but also efficiently.

After these combined sessions, BME students spend the remaining half of the semester in the BME teaching lab working on a small but real design project under the supervision of a BME professor. Generally, students form two-person teams and are tasked with building a small electronic device, such as a popcorn butter machine, a milk frother, a mini vacuum cleaner, a small cone and plate viscometer, and so on. Students usually spend one to two weeks conducting market analysis to better understand the existing products in the market, features of different products, price range, market demand, and market size, and so on. Students then start their own design, which needs to be different from what is available in the market. Students are allowed to use design/modeling software (such as AutoCAD), 3D printer, laser cutter, CNC machine, and other prototyping tools available in the teaching lab to make a physical product. Students have 5 weeks to achieve all their design goals and make a working prototype. At the end of the semester, all the devices made by the student teams are tested for their functionality. Design successes, failures, and the challenges students face during the design/prototyping process are discussed in class and documented in students’ written reports.

### EXT488 Part II: Shared Clinical Experiences (Spring, Year 1)

In this one-credit course (one 2-hour meeting per month, and 40+ hours of clinical shadowing per semester), BME students join the medical students for shared clinical experiences in various clinical locations (e.g., emergency department, pediatric urgent care, hospital biomedical engineering, operating room, labor and delivery, medical school clinical simulation center, mobile stroke unit ambulances, etc.). To be granted access to the clinical setting, BME students are required to go through a hospital clearance and orientation process, including verification of immunizations and titers, privacy and confidentiality training, and workplace safety training. In addition to joining the medical students, BME students also shadow hospital BME specialists, nurses, respiratory therapists, radiation technicians, paramedics, nursing assistants, and physicians. During these clinical experiences, BME students are expected to brainstorm and create at least three clinical need statements per shadow experience. Medical students, BME students, and the clinical faculty meet as a large group once a month to discuss and refine the clinical needs statements that are generated. At the end of the semester, BME students are expected to have a list of clinical needs they identified, which they then narrow down to three urgent or significant clinical needs.

At the end of the spring semester, BME students in the course series are expected to know how to conduct clinical observation, how to communicate effectively with clinicians, how to write clinical needs statements, how to translate a clinical need into an engineering problem, and how to evaluate the engineering feasibility of a solution through rapid prototyping. Equipped with these skills, BME students are ready to move on to the Summer Clinical Immersion Program.

### EXT488 Part III: Biodesign Summer Clinical Immersion (Summer, Year 1)

In this zero-credit course, BME students further narrow down the final three clinical needs they identified during EXT488 Part II and begin early-stage design and prototyping (with the support from BME, clinical and industry mentors). The overall objectives of this course include the following:

- Through direct clinical observation and consultation with clinicians and industry mentors, students are expected to identify one significant clinical need.
- Through a rapid prototyping process, students are expected to identify potential engineering solutions to a given clinical problem.
- Through literature search, intellectual property analysis, and consultation with industry mentors, students are expected to understand market needs and regulatory pathways associated with a medical innovation product.

All students receive a $2,000 stipend for participating in the Summer Clinical Immersion Program (20 hours per week). The typical schedule for this 6-week course is depicted in Table 1. During week 6 of the summer program, students make a pitch for their clinical need and initial proposed solution. Based on the clinical problem, the potential solution, the market potential of the product, and the intellectual property potential of the invention, all mentors (BME, clinical, and industry mentors) vote if a summer immersion project should be carried forward as a BME senior design project.

**Table 1:** The 6-week summer immersion program schedule

	Monday	Tuesday	Wednesday	Thursday	Friday
Week 1	Lectures: market analysis, product search, and patent search	Open discussion with clinical mentors	Open discussion with industry mentors	Research	Research
Week 2	Problem selection discussion with BME mentors	Problem selection discussion with clinical mentors	Problem selection discussion with industry mentors	Research	Finalize problem/project selection
Week 3	Clinical rotation and shadowing; discussion with all mentors (by individual appointments), research, and start design and prototyping.				
Week 4	Research, design, and prototype. Consult all mentors.				
Week 5	Regroup with BME mentors	Regroup with clinical mentors	Regroup with industry mentors	Draft a plan	Pitch the problem and solutions to all mentors; receive feedback
Week 6	Research based on feedback	Plan with BME mentors	Plan with clinical mentors	Plan with industry mentors	Final report due

### BME440/441: BME Senior Design Capstone Project Courses (Fall and Spring, Year 2)

During these two semesters, BME students are required to build a working prototype that addresses their self-derived clinical need. BME students who participated in the preceding biodesign internship program, and therefore had identified, screened, and performed an initial stakeholder and market analysis suggesting the idea was viable, are encouraged to find classmates interested in their project. In general, 1-2 students from the clinical immersion program find 2-4 other classmates to work with, and naturally take the leadership role of these design teams. This group of BME students are then joined by 2-3 medical students (who were in EXT 488 with the BME students), who act as near-peer consultants and assist the team’s clinical faculty mentors. In this capacity, medical students are the first in line in answering clinical questions and provide feedback about possible solutions to the BME students. In the event the medical students are unable to provide an answer or feedback, the question then goes to the clinical faculty mentor. During the whole senior design process, students stay in contact with the industry mentor for market and customer related questions.

## Reflection

**Fig. 1:**
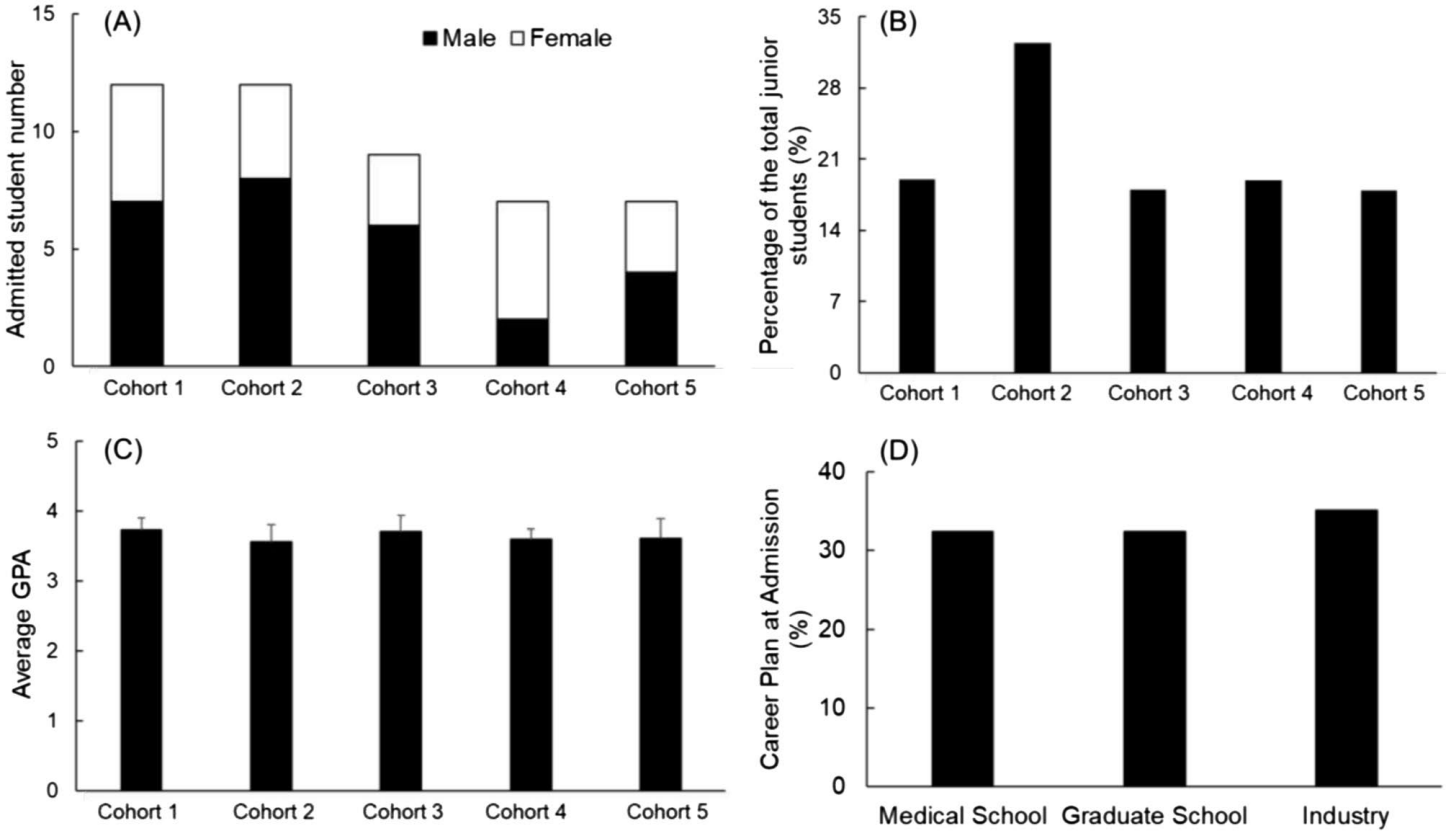
General information of the students that were admitted into the biodesign internship program. **A** The total number (male and female) students admitted into the program. **B** The percentage of the admitted students to the total number of BME juniors in the same year. **C** The average GPA of admitted students. **D** The overall distribution of student career goals at the time of admission

The first five cohorts of our longitudinal, interdisciplinary clinical immersion program included a total of 47 BME students. Figure 1 depicts the demographics of these students.

For EXT488 Part I, students provided their feedback through their end of the semester reports. Students were asked to 1) rate their overall experience on a 1-10 scale (1 being poor and 10 being very good); 2) summarize what they have learned from the experience and if it is helpful; and 3) if they have any recommendations and suggestions for the course instructors. The representative comments from students are shown in Table 2. Students overall enjoyed interacting with medical school faculty/students and industry mentors and were excited about hands-on and project-based learning. Many of them recognized a better understanding of the complexity of the medical device innovation process. Constructive feedback generally included students wanting additional time dedicated to group meetings and prototyping. The average students’ ratings ranged from 8.2±0.75 to 9.0±1.15 (mean±standard deviation). The lowest rating occurred in fall 2020, when the program was offered remotely during the pandemic.

**Table 2:**
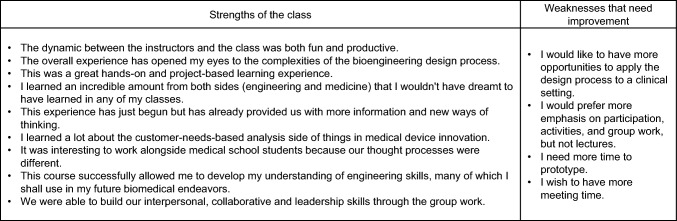
Representative comments from students on EXT488 – fall semester

In addition, from an instructor standpoint, because both the medical students and the BME students had busy academic schedules, it was challenging to find a time that both groups could come together. The importance of documentation practices to provide clear evidence of inventorship became apparent (especially in the later part of EXT488 and during senior design). A session was added at the beginning of the program (EXT488 Part I) in which the University’s Intellectual Property Partners’ Office joined the students to explain best practices for IP documentation.

For EXT488 Part II, students provided details of their clinical observation sessions in their end of the semester reports (time, location, clinicians shadowed, observation summary, clinical needs identified, etc.). Students were also asked to summarize what they have learned through the experience, and if they have any recommendations/suggestions for additional resources and support. Some representative student feedback from EXT488 Part II includes: “I found the semester’s work to be a rewarding experience”; “I learned a lot about the medical field from this internship that will forever help me in my career path as a biomedical engineer”; “This was a phenomenal experience thus far and I gained a ton of new information about how hospitals run and how the OR and ER operate”; “I feel like I learned a lot during this semester, and the most valuable part for me was the in-person shadowing”; “It is difficult to come up with clinical needs simply by thinking about them”; and “I cannot learn these knowledge from the textbook and I am really thankful.” All participating BME students were able to identify more than one viable clinical need through the shared clinical experiences.

Of 12 BME students per cohort who successfully completed EXT488 Part I and II, about 5-8 BME students per cohort chose to continue in the sequence and participate in Part III, the Biodesign Summer Clinical Immersion. Although students who participated received a $2,000 stipend as part of the R25 grant, it was not sufficient to cover the full cost of summer housing or living expenses, and because of this, some students were unable to continue in the program. Table 3 summarizes the participation rate in EXT488 Part III, the need statements generated, and the percentage of summer projects that progressed to BME senior design capstone projects. The most common reason for students not to participate in the summer immersion program was the high on-campus housing cost during the summer. During the pandemic, health concerns also prevented some students from participating. No quantitative feedback from the students was collected in EXT 488 Part II or Part III. This is a limitation of our study, and we would like to include it in future.

**Table 3:**
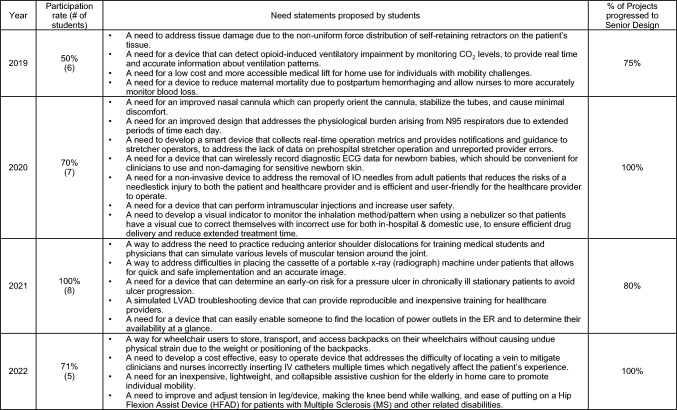
Summer immersion program participation rates, need statements, and percentages of projects proceeded to senior design

In total, 20 clinical needs generated and refined in EXT488 went on to be used as senior design project ideas. These ideas were worked on not only by BME and medical students participating in the longitudinal, interdisciplinary clinical immersion program, but also by approximately 60% of all BME students per graduating class.

In addition to the positive impact of this program on students’ hands-on learning and BME senior design capstone experience, other products of this program include conference abstracts/presentations, peer-reviewed journal publications, provisional patents/patents, and design competition awards (Table 4). Achieving milestones such as filing provisional patents was exceedingly rare prior to this program. It would seem as though the early collaboration between BME and medical school faculty, as well as the support from industry mentors, enabled students to identify significant clinical needs based on their own observation, and engaging in this process before their senior year certainly helped students to achieve more milestones during their overall design process.

**Table 4:**
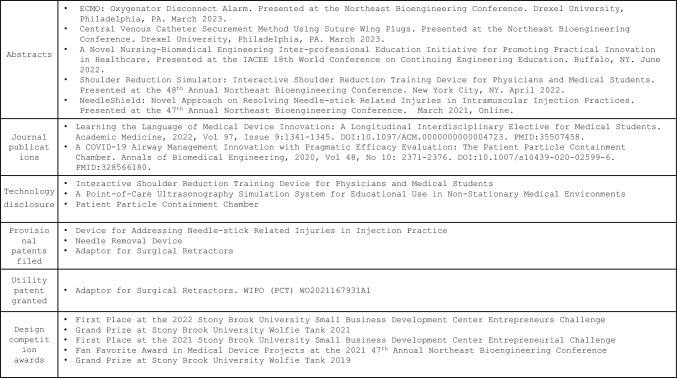
Other products of the program

Previous studies have demonstrated the importance of interdisciplinary collaboration between BME students, medical students, and clinicians, during the biodesign process [8–10]. Using similar approaches, our program provides an effective way to engage students in hands-on learning that starts with a real clinical need. The unique contents of this course sequence include the following: 1) this program provides BME students with unique opportunities to work directly with clinical mentors and medical students, starting from the junior year and continuing till the end of their senior year (a longitudinal process); 2) the clinical shadowing, which gives BME students an opportunity to identify clinical needs from their own experience; and 3) the summer clinical immersion program, which gives students an opportunity to work closely and extensively with their mentors (especially clinical and industry mentors) to validate the clinical needs they identify. These unique program contents are expected to enhance students' biodesign skills in communication (with clinicians), needs finding and screening, problem validation, and prototyping.

There are some limitations with the program in its current format. The program aimed to promote BME students’ learning of critical skills, e.g., communication, teamwork, research and prototyping, understanding intellectual property and market potential, and so on, needed for the future medical device innovation workforce development. Table 2 demonstrates some qualitative feedback we received from students, which might not be sufficient to assess our program outcomes. When the program was launched, three high-level goals were established. To effectively assess whether these goals are being achieved, they may need to be broken down into measurable indicators. For example, for goal 1 (BME students learn the shared language of medical device innovation with medical students via longitudinal process), we may use several indicators, including quality of the small collaborative projects in EXT488 Part I, BME students - medical students’ total contact time (both in and out of class over two years), the depth of medical students’ involvement in BME senior design projects, and BME students’ feedback, to assess the effectiveness of BME students’ learning through the longitudinal process. To assess if BME students have learned different aspects of biodesign and innovation before senior design (goal 3), students’ reports for the summer immersion program, especially the market analysis and IP evaluation sections, can be used. Additionally, students’ self-evaluations for EXT488 Part II and III can be collected for qualitative assessment, using structured analysis and thematic coding. We may need to revise our original goals to better meet the learning needs of the students.

The program in the current format does not have the capacity to accommodate all interested BME students. We plan to bring partners from more disciplines (in healthcare programs) to the biodesign process, and develop new courses, programs, and internship opportunities, to scale up participation and enhance the impact of our educational effort. We also would like to incorporate global collaborations to address healthcare challenges in low-resource environments.

Another limitation is that we do not have data to assess the long-term impact of the program on BME students’ career development. In our future studies, a long-term data collection plan will be needed to follow up with EXT488 students after they graduate.

## Human Subject Consideration

This study is approved for exemption, determined by IRB at Stony Brook University.

## Data Availability

Not applicable.
